# When Influenza Masquerades as Gastroenteritis: Acute Diarrhea in an Adult With Influenza B

**DOI:** 10.7759/cureus.94675

**Published:** 2025-10-15

**Authors:** Jimmy Joseph

**Affiliations:** 1 Internal Medicine, Aster DM Healthcare, Dubai, ARE

**Keywords:** acute gastroenteritis, acute undifferentiated febrile illness, diarrhea, fever, influenza a, influenza b, oseltamivir

## Abstract

Influenza is a common viral respiratory illness caused by influenza A and B viruses, typically presenting with fever, cough, sore throat, myalgia, and malaise. Gastrointestinal symptoms are less common, particularly in adults, but may occasionally predominate. Diarrhea as the initial manifestation of influenza is rare and may mimic acute gastroenteritis, leading to diagnostic delay and inappropriate therapy.

This is a case report of a 46-year-old male with influenza B presenting primarily with acute watery diarrhea and fever, without initial respiratory symptoms. The atypical presentation initially led to a misdiagnosis of acute gastroenteritis. Confirmation of influenza B was obtained via rapid antigen testing, and the patient was successfully treated with oseltamivir, achieving full recovery.

This case underscores the importance of considering influenza in the differential diagnosis of febrile diarrheal illness, especially during seasonal epidemics and in travelers. Recognition of such atypical presentations enables early laboratory confirmation and timely antiviral therapy, preventing unnecessary antibiotic use, and contributes to improved clinical and public health outcomes.

## Introduction

Influenza is an acute, highly contagious viral illness caused by influenza viruses A and B, belonging to the Orthomyxoviridae family. Seasonal influenza affects millions worldwide each year, with an estimated one billion infections, three to five million cases of severe illness, and up to 650,000 respiratory-related deaths annually, according to the World Health Organization [1]. The disease typically presents with abrupt onset of fever, chills, cough, sore throat, rhinorrhea, myalgia, arthralgia, and malaise, reflecting the primary tropism of the virus for the respiratory tract [2,3].

Although respiratory features dominate, systemic and extrapulmonary manifestations are increasingly recognized. Gastrointestinal (GI) symptoms, including nausea, vomiting, and diarrhea, have been reported across multiple influenza epidemics [4,5]. These symptoms are classically more frequent in children but may occur in adults as well. Importantly, gastrointestinal presentations can obscure the diagnosis, leading to misclassification as acute gastroenteritis, food poisoning, or tropical infections such as dengue fever in endemic regions [6].

The prevalence of GI involvement in influenza is variable. A meta-analysis by Charu et al. found that diarrhea occurred in 6% of influenza A cases and 10% of influenza B cases [7]. Other studies confirm that children are more likely to develop diarrhea (10-15%), whereas adults demonstrate lower prevalence (2-6%) [8,9]. Notably, influenza B has been linked to greater gastrointestinal tropism than influenza A [7,10].

Pathophysiologically, influenza virus has been detected in stool samples, raising the possibility of intestinal replication [11]. However, the majority of symptoms are believed to result from systemic cytokine release and immune-mediated inflammation rather than direct mucosal infection [12]. Elevated interleukin-6 and tumor necrosis factor-α have been associated with gastrointestinal features, further supporting an immunological mechanism [13].

Atypical influenza presentations pose a diagnostic challenge. In adults, diarrhea without respiratory symptoms may initially mimic bacterial gastroenteritis, viral enteritis, or tropical febrile illnesses such as malaria or dengue [6,14]. This is particularly important in travelers or in regions with overlapping seasonal epidemics. Misdiagnosis can delay the initiation of antiviral therapy and increase unnecessary antibiotic usage, both of which were initially seen in our patient. Furthermore, atypical presentations may contribute to underreporting and underrecognition of influenza burden in surveillance systems [15].

The clinical relevance of recognizing atypical influenza is significant. Timely initiation of neuraminidase inhibitors such as oseltamivir is known to shorten the duration of symptoms, reduce complications, and lower hospitalization risk when commenced within 48 hours of onset [12]. Awareness of gastrointestinal-predominant influenza presentations enables clinicians to broaden differential diagnoses in febrile diarrheal illness, particularly during influenza season or outbreaks.

This report describes a 46-year-old male who developed acute watery diarrhea and fever as the initial presentation of influenza B.

## Case presentation

A 46-year-old Indian male, previously healthy, presented with a one-day history of high-grade fever and watery diarrhea. The diarrhea was described as small-volume, watery in consistency, and occurred two to three times within 24 hours. It was associated with nausea but without vomiting, no abdominal pain, and no blood in the stool. He denied cough, rhinorrhea, chest pain, or dyspnea at the time of presentation. The patient had recently returned from India two days prior, and there was no history of similar illness among family members or contacts.

On examination, he was alert and oriented. Vital signs revealed a temperature of 38.8 °C, pulse 103/min (regular), blood pressure 128/80 mmHg without postural hypotension, and respiratory rate 20/min with oxygen saturation of 98% on room air. He appeared mildly dehydrated, with a dry tongue, but skin turgor and capillary refill were normal. Cardiovascular, respiratory, and abdominal examinations were unremarkable.

Laboratory evaluation (Table 1) on day one demonstrated hemoglobin 15.3 g/dL (reference: 13-17 g/dL), total leukocyte count 5.33 ×10⁹/L (4-11 ×10⁹/L) with a differential of neutrophils 68% (40-75%) and lymphocytes 13% (20-45%)- lymphopenia, platelets 192 ×10⁹/L (150-400 ×10⁹/L), and C-reactive protein (CRP) 2.23 mg/L (<5 mg/L). Stool microscopy was normal, with no leukocytes, ova, or cysts. Based on the initial picture, a working diagnosis of acute gastroenteritis was made, and the patient was managed with intravenous fluids, oral rehydration solution, intravenous and oral paracetamol, tab cefixime 400 mg once daily, and supportive care.

**Table 1:** Laboratory Investigations WBC- White blood cell count, CRP- C Reactive Protein

Parameter	Day 1	Day 2	Reference Range
Hemoglobin	15.3 g/dL	–	13–17 g/dL
WBC	5.33 ×10⁹/L	–	4–11 ×10⁹/L
Neutrophils	68%	–	40–75%
Lymphocytes	13%	–	20–45%
Platelets	192 ×10⁹/L	–	150–400 ×10⁹/L
CRP	2.23 mg/L	–	<5 mg/L
Stool microscopy	Normal	–	–
Dengue NS1 antigen	–	Negative	–
Malaria parasite	–	Negative	
Strep throat antigen	–	Negative	–
Throat culture	–	Sterile	–
Influenza A rapid antigen	–	Negative	–
Influenza B rapid antigen	–	Positive	–

On the following day, he re-presented with persistent fever of 39 °C and ongoing diarrhea (two episodes in 24hours). He additionally complained of new systemic features including mild throat discomfort for 12 hours, generalized body aches, joint pains, myalgia, and headache. He continued to deny respiratory symptoms such as cough or rhinorrhea. Vitals remained stable, and systemic examination was unchanged. Repeat laboratory evaluation on day two included dengue NS1 antigen (negative), malaria parasite (negative), rapid streptococcal antigen test (negative), and throat swab culture (sterile). A nasopharyngeal swab for influenza testing revealed influenza B positive and influenza A negative on rapid antigen assay.

Based on these findings, the diagnosis was revised to influenza B infection presenting with atypical gastrointestinal manifestations. The patient was commenced on oseltamivir 75 mg orally twice daily for five days. His clinical course was favorable: diarrhea resolved within 48 hours of initiating antivirals, fever subsided by the third day, and he achieved complete recovery without complications by day seven.

## Discussion

Influenza is widely recognized as an acute respiratory viral infection characterized by fever, cough, sore throat, myalgia, and malaise [2]. While systemic symptoms such as headache and fatigue are common, GI manifestations are less frequently observed, particularly in adults. Our case illustrates an atypical presentation of influenza B in which watery diarrhea and fever were the initial and predominant symptoms, mimicking acute gastroenteritis and delaying recognition of the underlying viral etiology.

Gastrointestinal symptoms have been increasingly reported during seasonal and pandemic influenza outbreaks. In a study from the 2009 H1N1 pandemic, 25% of hospitalized patients experienced nausea, vomiting, or diarrhea, although diarrhea alone was less frequent [4]. Minodier et al. confirmed that while GI manifestations are not universally present, their prevalence is significant enough to warrant attention [5]. A meta-analysis estimated diarrhea prevalence at approximately 6% in influenza A and 10% in influenza B, with higher rates in pediatric populations [7]. Other reports indicate that up to 15% of children experience diarrhea during influenza illness, compared to 2-6% of adults [8,9]. These findings are consistent with our case, highlighting diarrhea as an uncommon but important atypical presentation in adults.

Evidence suggests that influenza B may have a greater predilection for gastrointestinal involvement than influenza A [7,10]. Several factors may contribute to this difference. Detection of viable influenza virus in stool has been demonstrated, supporting the possibility of intestinal replication [11]. Additionally, the immune response to influenza B may induce higher levels of systemic cytokines, including interleukin-6 and tumor necrosis factor-α, which can increase intestinal permeability and alter motility [12,13]. These mechanisms may explain the patient’s watery diarrhea, despite the absence of typical respiratory features at initial presentation.

The clinical overlap between influenza and other febrile illnesses complicates diagnosis, particularly in tropical and subtropical regions where dengue, malaria, and bacterial gastroenteritis are prevalent [6,14]. This patient had recently traveled from India, a setting where dengue and enteric infections are common. Initial investigations, including dengue NS1 and stool cultures, were appropriately performed. However, the absence of classical respiratory symptoms contributed to the initial misdiagnosis of acute gastroenteritis and empiric antibiotic use. This reflects a common challenge: influenza without respiratory involvement is often overlooked, leading to delayed or missed diagnosis.

This case also underscores the importance of diagnostic vigilance in preventing inappropriate antimicrobial use. Empirical antibiotics such as cefixime were initiated under the assumption of bacterial gastroenteritis, yet laboratory evaluation showed no inflammatory markers or stool abnormalities. Studies have demonstrated that misdiagnosis of viral illnesses as bacterial infections contributes significantly to unnecessary antibiotic prescribing, fueling antimicrobial resistance [6]. Recognition of influenza in atypical settings can therefore improve antibiotic stewardship by reducing inappropriate prescriptions.

Timely initiation of neuraminidase inhibitors remains critical to improving patient outcomes in influenza. Evidence from randomized controlled trials and systematic reviews demonstrates that oseltamivir, when started within 48 hours of symptom onset, shortens symptom duration, reduces complication rates, and lowers hospitalization risk [12]. In our patient, oseltamivir led to rapid resolution of diarrhea within 48 hours and defervescence by day three. This favorable response reinforces the importance of maintaining clinical suspicion for influenza even in the absence of respiratory features, as delayed recognition could have led to prolonged illness and unnecessary antimicrobial exposure.

Atypical influenza presentations also carry public health implications. Influenza surveillance systems rely heavily on respiratory symptom-based case definitions, which may underrepresent cases with gastrointestinal or systemic-dominant presentations [15]. This underreporting may underestimate the true burden of disease and impair timely outbreak detection. Greater awareness among clinicians of influenza’s atypical manifestations can improve surveillance accuracy, guide infection control measures, and inform vaccination strategies.

In summary, this case highlights acute diarrhea as an unusual manifestation of influenza B in adults. The absence of respiratory features delayed recognition and led to empiric antibiotic use. Awareness of atypical influenza presentations, particularly gastrointestinal-dominant cases, is vital during epidemic seasons and in travelers from endemic regions. Clinicians should maintain a broad differential diagnosis for febrile diarrheal illness, incorporate influenza testing when appropriate, and initiate antivirals promptly once confirmed. Such vigilance improves patient outcomes, reduces unnecessary antimicrobial use, and strengthens public health surveillance.

## Conclusions

Influenza is usually a respiratory illness, but atypical gastrointestinal presentations may occur. Diarrhea, while more common in children, is rare in adults and may obscure the diagnosis. Our case of influenza B presenting as acute diarrhea emphasizes the need for clinicians to consider influenza in febrile diarrheal illnesses, especially during epidemic seasons or after travel. Early testing and prompt antiviral initiation not only improve patient recovery but also reduce unnecessary antibiotic use and contribute to accurate disease surveillance. Recognizing atypical influenza presentations remains essential for effective clinical and public health management.
